# Modularity and evolutionary constraints in a baculovirus gene regulatory network

**DOI:** 10.1186/1752-0509-7-87

**Published:** 2013-09-04

**Authors:** Juliana Velasco Oliveira, Anderson Fernandes de Brito, Carla Torres Braconi, Caio César de Melo Freire, Atila Iamarino, Paolo Marinho de Andrade Zanotto

**Affiliations:** 1 Department of Microbiology, Institute of Biomedical Sciences – ICB II, Laboratory of Molecular Evolution and Bioinformatics, University of São Paulo – USP, São Paulo, SP, Brazil; 2 Departamento de Microbiologia, Instituto de Ciências Biomédicas - ICB II, Laboratório de Evolução Molecular e Bioinformática, Universidade de São Paulo - USP, Av. Prof. Lineu Prestes, 1374, São Paulo CEP: 05508-900, Brasil; 3 Laboratório Nacional de Ciência e Tecnologia do Bioetanol (CTBE), Centro Nacional de Pesquisa em Energia e Materiais (CNPEM), Campinas Caixa Postal 6170, 13083-970, Brazil

**Keywords:** Baculovirus, Transcriptome, Real-time PCR, Gene regulatory network, Overlapping transcripts, Modularity

## Abstract

**Background:**

The structure of regulatory networks remains an open question in our understanding of complex biological systems. Interactions during complete viral life cycles present unique opportunities to understand how host-parasite network take shape and behave. The *Anticarsia gemmatalis* multiple nucleopolyhedrovirus (AgMNPV) is a large double-stranded DNA virus, whose genome may encode for 152 open reading frames (ORFs). Here we present the analysis of the ordered cascade of the AgMNPV gene expression.

**Results:**

We observed an earlier onset of the expression than previously reported for other baculoviruses, especially for genes involved in DNA replication. Most ORFs were expressed at higher levels in a more permissive host cell line. Genes with more than one copy in the genome had distinct expression profiles, which could indicate the acquisition of new functionalities. The transcription gene regulatory network (GRN) for 149 ORFs had a modular topology comprising five communities of highly interconnected nodes that separated key genes that are functionally related on different communities, possibly maximizing redundancy and GRN robustness by compartmentalization of important functions. Core conserved functions showed expression synchronicity, distinct GRN features and significantly less genetic diversity, consistent with evolutionary constraints imposed in key elements of biological systems. This reduced genetic diversity also had a positive correlation with the importance of the gene in our estimated GRN, supporting a relationship between phylogenetic data of baculovirus genes and network features inferred from expression data. We also observed that gene arrangement in overlapping transcripts was conserved among related baculoviruses, suggesting a principle of genome organization.

**Conclusions:**

Albeit with a reduced number of nodes (149), the AgMNPV GRN had a topology and key characteristics similar to those observed in complex cellular organisms, which indicates that modularity may be a general feature of biological gene regulatory networks.

## Background

Cellular pathways and gene regulatory networks (GRNs) are complex systems that emerge under natural selection and bare distinct evolutionary proprieties such as modularity. Modularity is an effective mechanism for keeping perturbations confined while preserving the complete system [1]. It is widely observed in various organisms and is possibly a fundamental biological design principle [2-4]. Emergent proprieties in networks also impose constraints on individual genes, mostly due to epistasis, leading to gene conservation. Important enzymes and other essential proteins – as reported by flux balance analysis [5,6] – tend to vary less than those under lower functional load [7], indicating that the flow of matter through metabolic networks expounds an evolutionary constraint imposed on components of any given pathway. These findings where confirmed for yeast, in which highly connected enzymes evolve more slowly than less connected ones [8]. Viruses intertwine their metabolic functions with those of the host cell, which allows infected cell to be understood as superorganisms, comprising the minimal essential condition for viral replication and biomagnification [9]; yet how viruses organize their gene network remains an open question.

Baculoviruses provide a rich environment where to investigate these relations, since these viruses have many completely sequenced genomes, each one encoding around one hundred plus open reading frames (ORFs) with transcription strategies and defined overall temporal regulation. The *Baculoviridae* constitute a family of invertebrate viruses that infect mainly insects of the order Lepidoptera, with large circular, covalently closed, double-stranded DNA genome [10,11]. The analysis of fifty-seven complete baculovirus genomes has shown 37 genes probably shared among all of them [12-17], most of which are involved in essential processes, such as replication, transcription and oral infectivity. The *Anticarsia gemmatalis* multiple nucleopolyhedrovirus (AgMNPV) has been used in Brazil and other countries to control the velvet bean caterpillar *Anticarsia gemmatalis* (Lepidoptera: Noctuidae), an important pest of soybean crops [18,19]. The prototype AgMNPV isolate 2D (AgMNPV-2D) [20,21] has a genome of 132,239 bp, which may encode 152 ORFs [22].

Gene expression and replication cycle of baculovirus appear to be regulated by viral factors and the cellular milieu, where distinct gene classes are thought to be trans-activated (directly or indirectly) by closely related transcription complexes, causing expression to be controlled by distinct promoters, activated in a temporal concerted fashion during the infection cycle [10]. Three main transcriptional phases can be distinguished: *early* (immediate early and delayed early), *late* and *very late*[10]. The early phase precedes the onset of viral DNA replication and includes transcription of genes involved in host modulation, viral DNA replication and regulation of delayed early and late gene expression. The late and very late phases follow DNA replication and include the expression of genes required for virus assembly and occlusion (structural genes) [23].

An interesting feature of baculovirus transcription is that some ORFs can be transcribed in tandem, spanning up to seven units [24-26]. Specific genomic regions encode a wide variety of transcripts with different lengths, the so-called ‘overlapping transcripts’ [27-29]. Several tandem transcripts were mapped to different genomic regions of *Autographa californica* nucleopolyhedrovirus (AcMNPV) [27,30] and *Bombyx mori* nucleopolyhedrovirus (BmNPV) genomes [31]. The expression profiles of six AgMNPV genes were investigated: *egt*[32]; *p10*[33]; *v-trex*[34]; *helicase*[35]; *iap-3*[36] and *p74*[37]. Nevertheless, there was no comprehensive study done so far on its complete gene content transcription and regulation during the replication cycle. Therefore, it is of interest to address the role of gene expression regulation in shaping baculovirus genome organization and evolution. Here we analyzed transcriptional organization of AgMNPV compared to randomized datasets in two distinct cell lines with different infection kinetics [38]. Our analysis focused on: (*i*) the viral transcriptome profile, (*ii*) the structural properties of its gene regulatory network (GRN), (*iii*) genomic arrangement of transcripts of distinct viral cycle phases and, (*iv*) association of transcripts to distinct temporal promoter types.

## Results

### Viral genes are expressed sooner and at higher amounts in its permissive cell line

**Figure 1:**
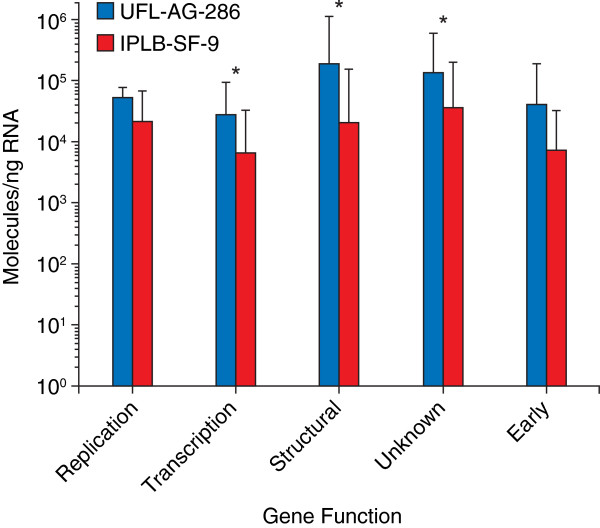
**Relative differences of mRNA expression during infections in UFL-AG-286 and IPLB-SF-9 cell lines.** Median values of mRNA expression of genes associated from left to right: **(*i*)** DNA replication, **(*ii*)** transcription, **(*iii*)** structural proteins, **(*iv*)** genes with unknown function and, **(*v*)** early genes. The asterisk (*) indicates gene groups that had significantly difference between cell lines (*p<*0.005).

We performed a series of quantitative real-time PCR experiments to determine the expression profile of AgMNPV-2D 152 predicted ORFs [22], following infection of two permissive cell lines with different infection kinetics [38] at 0, 1, 3, 5, 7, 9, 11, 13, 24 and 48h post infection (p.i.). UFL-AG-286 was isolated from the natural host of AgMNPV [39], and IPLB-SF-9 was established from a different Noctuidae genus [40]. All pairs of amplicons for each gene were successfully amplified, sequenced and verified (see Methods). Except for ORFs 64 and 83, all remaining 149 ORFs were expressed in both cell lines (ORF 135 was excluded from the analysis due to nonspecific amplification). To help inspection, the average values (log 10) from three independent experiments are shown in Additional file 1: Table S1 (UFL-AG-286 cell line) and Additional file 2: Table S2 (IPLB-SF-9 cell line). Several ORFs reached significant levels of expression at different time points, reinforcing the expectation of a temporal structure in the viral cycle. At 7 h p.i., most genes were detected in both cells. Nevertheless, genes were expressed earlier in UFL-AG-286 cells than previously reported in literature for most baculovirus, including AgMNPV genes previously studied using Northern blot and RT-PCR [32,33,35,36]. We compared the patterns obtained from the two cell lines considering the magnitude and the first detection (post-infection) of significant gene expression. Temporal differences in both cell lines were complex, with several ORFs being expressed at different relative times in each cell line. For instance, certain genes were expressed much later in the IPLB-SF-9 cell line (such as *odv-e27* (ORF 140)) and other genes were expressed in the same time post-infection (such as *lef-1* (ORF 19)), or with a delay of 1 h such as *cg30* (ORF 85), which was expressed a 0 h in UFL-AG-286 and 1 h latter in IPLB-SF-9. We observed that most genes were more expressed in the UFL-AG-286 cell line than in the IPLB-SF-9 (Figure 1), reaching in some instances more than fifty-fold increase (such as, *39k*/*pp31* (ORF 25)), with the exception of *pe38* (ORF 149) that was expressed at higher levels in IPLB-SF-9 cells. Furthermore, differences in expression intensity between cell lines decreased during infection, to the extent that at 48 h p.i., *ag4*, *ag20*, *ag44* and some others were more expressed in IPLB-SF-9 than in UFL-AG-286. In sum, the relative temporal pattern for several genes changed depending on the cell line (Additional file 1: Table S1 and Additional file 2: Table S2).

### The inferred gene regulatory network has non-random proprieties

Since we had three independent replicates of infection experiments lasting 48 hours each, we used a single 144 hrs time series to infer a putative AgMNPV-2D gene regulatory network (GRN) from 149 ORFs unfolded during infection in UFL-AG-286 and likewise, a single 144 hrs time series for IPLB-SF-9. Under the assumption of a recurrent temporal regulation program for viral gene expression, this approach should maximize true gene-to-gene cross-correlation signal present in the data, while mimicking three successive infection cycles in a row. Ranked lists of directed node interactions were generated with GENIE3 from expression data of AgMNPV-2D infecting UFL-AG-286, infecting IPLB-SF-9 and the respective randomized data (UFL-AG-286rand and IPLB-SF-9rand). One thousand links were necessary on average to assemble a single connected component with 149 nodes for all GRN (including those generated from random datasets). Nevertheless, the complete network for AgMNPV-2D on UFL-AG-286 needed 1120 edges to include all nodes into a single component, which was in agreement with similar but random GRNs, which had an average of 1030 edges. Preliminary exercises with random GRNs revealed that the data generated from our complete lists of directed node interactions generated with GENIE3 lacked essential complex features of known biological networks such as modularity. Since we needed more realistic null models to better discern features of real networks from the data, we decided to keep only the relevant links that would be sufficient to generate a single component network while generating randomized networks by shuffling the real data time points. This procedure obliterated the time structure of the real data but kept its value range and sampling density.

### The network architecture displays increasing modularity

**Figure 2:**
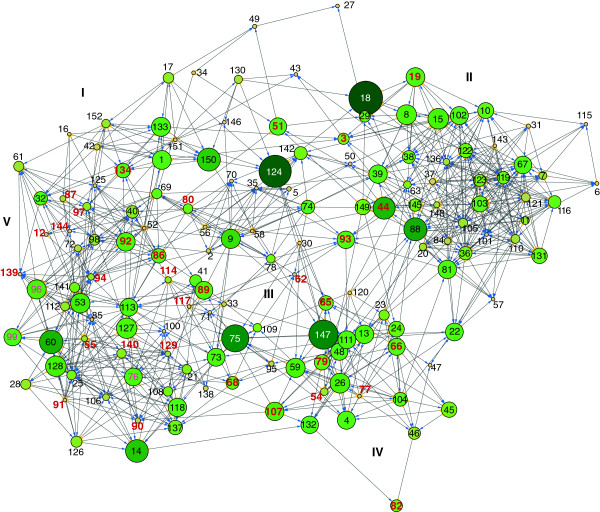
**Gene regulatory network (GRN) of AgMNPV-2D infecting UFL-AG-286 cells generated with Cytoscape v2.8.0.** A single component network shows the directed links (arrow edges) among 149 ORFs. Nodes (ORFs) have sizes proportional to their betweenness centrality (BC) and darkness proportional to stress coefficient (SC), both of which indicate node importance in the GRN. Five communities **(I**-**V)** were identified with the GLay clustering method, which indicate modules of highly interconnected nodes. Numbers correspond to ORFs in the AgMNPV-2D genome (see Additional file 1: Table S1) and the 37 genes shared among all baculoviruses are colored in red.

The ‘most important’ links were pruned using the maximum neighborhood component (MNC) and the density of maximum neighborhood component (DMNC) algorithms with *Hubba*, down to 765 (UFL-AG-286), 991 (IPLB-SF-9), 713 (UFL-AG-286rand) and 727 nodes (IPLB-SF-9rand), still maintaining a single large component yielding a ratio of around 5 edges per node. A model of the reduced AgMNPV-2D GRN in UFL-AG-286 cells (Figure 2) depicts few communities of highly interconnected nodes organized around a single large component, where the importance of links caused nodes to come closer together (force-directed layout). We inspected the topological features and attributes of this AgMNPV-2D GRN with the Network Analysis plug-in in Cytoscape and observed that the real data GRN had a trend for the increase of average clustering coefficient (ACC) distribution as the number of neighbors of a node increased, while a negative dependence tendency was observed on the scrambled expression data (Additional file 3: Figure S1). This trend was more pronounced in the infection in UFL-AG-286 cells (Additional file 3: Figure S1A and S1B). The average connectivity distribution of all neighbors of a node was also significantly different in real and scrambled data (Additional file 3: Figure S1). As the number of neighbors increased, real data GRN had a larger number of neighbors with higher ANC with positive increase of ANC (Additional file 3: Figure S1). We also observed that the GRN obtained infecting UFL-AG-286 differed from all others by having two nodes with stress centrality values one order of magnitude greater than that observed for both real and scrambled data: (*i*) *egt* (ORF 18) with SC = 1686 and, (*ii*) *gp64* (ORF 124) with SC = 1551, followed by *ag75* and *ag147*. Visual inspection of the force-directed GRN layout reveals that these four nodes appear to interconnect adjacent modules in the GRN (Figure 2). The GRN in IPLB-SF-9 shared several basic features with that unfolding in UFL-AG-286. However, for the sake of brevity details on the reduced modularity (possibly due to reorganization of the GRN under perturbation), will be dealt with elsewhere. Our results expounded a genuine modular architecture not caused by chance in the AgMNPV-2D GRN and they also indicated that this modularity was more pronounced in the infection in UFL-AG-286.

It is of utter importance to emphasize that node linkages in our GRNs depict the relationships *among regulatory elements* inferred from gene expression profile (transcriptional data). Hence, it has to be made clear that the networks we inferred convey the implied associations among transcription regulation elements *per se*, and not necessarily gene product function relationships (such as in an interactome). Fittingly, genetic regulatory functions are best understood when viewed in terms of *intergenic linkages of diverse modalities*, and these non-linear functions are not visible at the level of any individual gene [41].

### The gene communities (modules) have redundant functions

**Figure 3:**
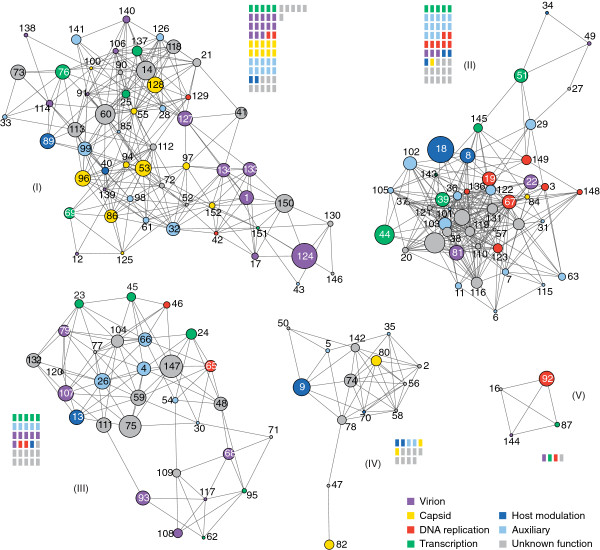
**Modular structure of AgMNPV-2D network unveiled with GLay clustering method.** The five different communities are ordered by their size and are populated with some particular distinctions. The comparative amount of gene function in each community is represented by colored rectangles. Community **I** is densely populated with subsets of nodes associated with structural functions; community **II** has subsets of nodes more related to DNA replication and auxiliary functions; community **III** is homogeneously populated with nodes from different functional categories; community **IV** includes diverse genes without assigned functions; and community V is the smallest, where *helicase* (ORF 92) is located. Nodes represent ORFs of AgMNPV-2D and have colors according to the functional classification based on prior knowledge about baculovirus genes. Nodes also have different size representing the importance (betweenness centrality) of the ORF in the complete GRN (see Figure 2).

We used the GLay clustering method in Cytoscape to detect ‘gene communities’ (modules) in the GRN. The modularity (*Q*) in UFL-AG-286 was 0.56 and we found 5 communities adding to a total of 662 edges. This value (*Q* = 0.56) was significant since it was higher than the 99% upper value for the confidence interval (*Q* = 0.50) obtained for the other GRNs (*Q* = 0.46 for UFL-AG-286rand, 0.41 for IPLB-SF-9 and *Q* = 0.46 for IPLB-SF-9rand). We then observed that each one of the five modules was populated differently when we ordered the ORFs in six functional categories [42,43]: virion and capsid (structural), DNA replication, transcription, host modulation or auxiliary (Figure 3). Community I gathered most of genes associated with structural functions (23 of 36; χ^2^ = 14.002, *p =* 0.0002; d.f. = 1) while, more than 85% of its nodes (48 of 56; χ^2^ = 27.572, *p<*0.0001; d.f. = 1) are conserved in almost all sequenced group I alphabaculoviruses (including 13 shared genes among all baculoviruses). Community II was the most densely interconnected module (χ^2^ = 7.030, *p=*0.008; d.f. = 1), having one third of its genes (15 of 45 nodes; χ^2^ = 16.659, *p <* 0.0001; d.f. = 1) also conserved among group I. This community had the larger number of nodes associated with DNA replication (7 of 12; χ^2^ = 4.900, *p =* 0.0269; d.f. = 1) and had slightly more (albeit statistically insignificant) members with auxiliary functions (13 of 30; χ^2^ = 3.073, *p =* 0.0796; d.f. = 1). Community III was homogeneously populated with nodes from different functional categories. Community IV consisted mainly of genes without assigned functions (8 of 14; χ^2^ = 4.689, *p=*0.0304; d.f. = 1). Community V had only four ORFs and one of them was the *helicase*. Interestingly, different functional classes were dispersed among different node communities, which is suggestive of redundancy.

### Gene function impacts on expression level variation and on GRN topology

**Figure 4:**
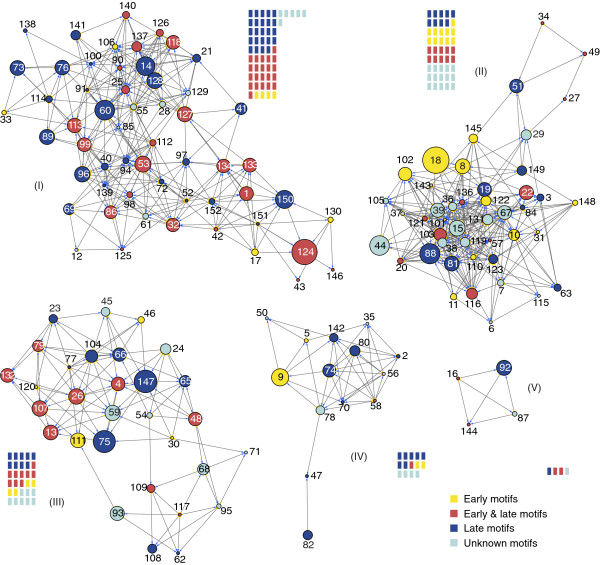
**Map of promoter types of AgMNPV-2D in UFL-AG-286 network.** Nodes represent ORFs of AgMNPV-2D and have colors according to the different promoter motifs (see text). The comparative amount of promoter motifs in each community is represented by colored rectangles. Community **I** is mainly populated by genes with late promoters motifs; community **II** is densely populated by ORFs expressed from unknown promoters; community **III**, **IV** and **V** have diverse promoter types. Nodes also have different size representing the importance (betweenness centrality) of the ORF in the complete GRN (see Figure 2).

We further investigated how the GRN architecture related to other features of nodes that were not considered during the inferential process. This is because the GRN we inferred is based on RNA expression through time and should reflect, to a greater extent, mostly the temporal program of gene expression. Therefore, given that promoter elements are known to have considerable temporal specificity, we first searched promoter motifs upstream of the initiation codon of each ORF and mapped as attributes in the five main communities (Figure 4). Within this region, 50 (33.56%) ORFs had a late motif, 23 (15.44%) contained an early motif, 42 (28.19%) had both early and late, and 34 (22.81%) lacked any recognized motif (Figure 4). The average clustering coefficient of early genes was higher for 20 genes with early promoters (ACC = 0.2195) than for 50 genes with only typical promoter elements (ACC = 0.1768), although not statistically different at a significance level of *α*≥ 0.05. Community I had mainly nodes associated with late motifs (45 of 56 nodes with late and early & late motifs; χ^2^ = 12.311; *p =* 0.0005; d.f. = 1) and a remarkable and significant absence of ORFs with upstream unique early promoters (5 of 56 nodes; χ^2^ = 40.898, *p* < 0.0001; d.f. = 1). Community II had a large number of genes with unknown promoters (15 of 45; χ^2^ = 4.047; *p =* 0.0442; d.f. = 1).

Given the apparent assortativity (that is, the preferential attachment of nodes by shared properties) of both gene function and promoter types, we then compared expression profiles among genes with correlated functions, such as: (*i*) replication: *dnapol*, *lef-1, lef-2* and *helicase;* (*ii*) transcription: *lef-4, lef-5*, *lef-8*, *lef-9, p47* and *vlf-1*; (*iii*) structural proteins: *pif-1, pif-2*, *gp41, odv-e56*, *odv-ec27, p74, p6.9*, *vp91*, *vp39*, *vp1054* and *p33;* (*iv*) core genes with unknown function; (*v*) some immediate-early genes that do not belong to the 37 conserved genes (*cg30*, *ie-0*, *ie-1*, *ie-2*, *me53* and *pe38*); and carried out the statistical analysis (ANOVA) among groups followed by Tukey’s multiple comparison test, assuming a significance level at *p <* 0.05. We noticed that early genes and those associated with DNA replication had less difference in their temporal and quantitative profiles between the two cell lines (Figure 1). This result suggested that core conserved functions do appear to have more invariance in the temporal structure of their expression program. Crucially, this temporal consistency was also associated with a trend on the association of a key GRN topologic feature and gene variability, since core functions (replication and capsid) had a higher median betweenness centrality (BC = 0.0019, Additional file 4: Figure S2) with a large variance and a significantly lower genetic diversity (θ = 0.42 ± 0.035), while satellite (all the others) functions had lower median betweenness centrality (BC = 0.0013) with large variance and a significantly higher genetic diversity (θ = 0.47 ± 0.044, Additional file 4: Figure S2).

### The spatial organization of regions of overlapping transcription (ROTs) is important and conserved

To better infer the relation between time expression and gene position, we built a syntenic map for the complete genome of thirteen Group I alphabaculoviruses (Additional file 5: Figure S3 and Additional file 6: Table S3) with twenty-two highly conserved ‘regions of overlapping transcription’ (ROTs) defined as multiply transcribed genomic loci encoding tandem RNA transcripts of different lengths. These ROTs had almost the same overall order and relative orientation in all genomes, even when genomic reorganization events, such as inversions and gene deletions took place. When compared to other genomic regions, for all 13 genomes investigated, we found in ROTs a higher frequency of ORFs with very short or absent intergenic spacers (overlapping genes) (χ^2^ = 65.041, *p* < 0.00001; d.f. = 1). If ROTs entail the synthesis of overlapping transcripts in alphabaculoviruses, it is possible that approximately 40% of the AgMNPV-2D genes (61 of 152) could be transcribed in tandem.

**Figure 5:**
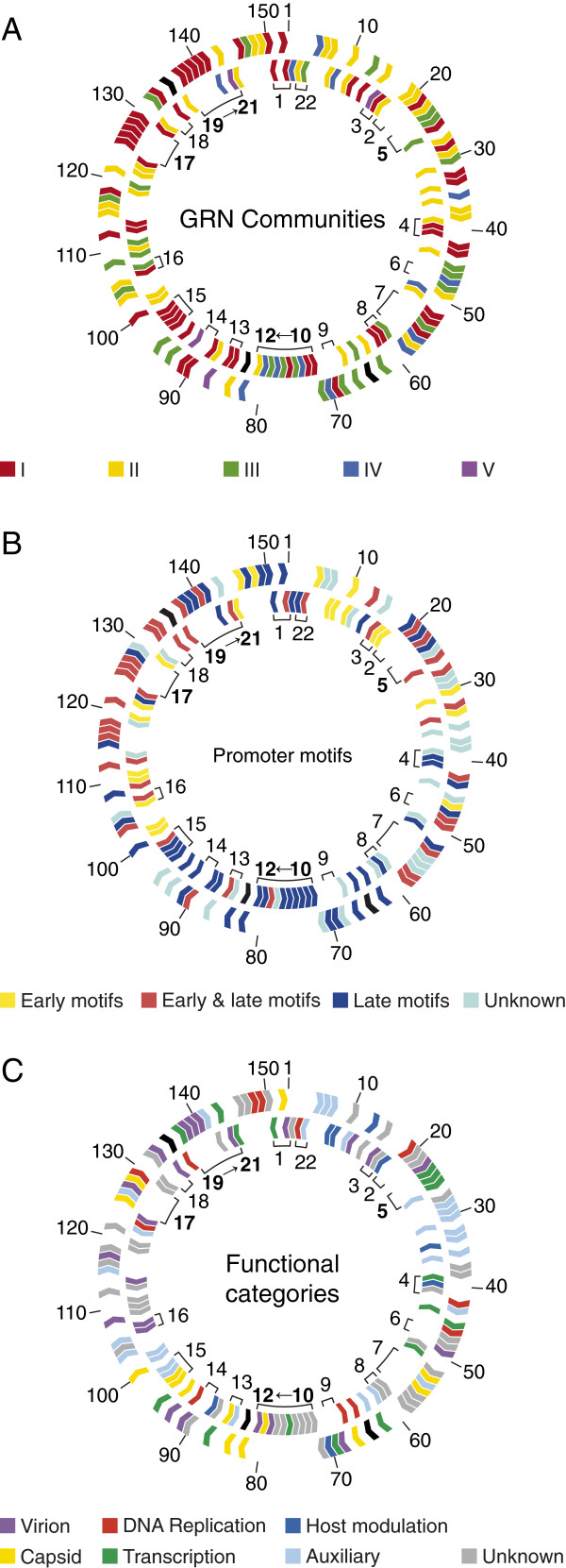
**Physical and functional organization of the AgMNPV-2D genome.** The outer and the inner ring represent ORFs in positive and negative sense, respectively. The outer numbering means the AgMNPV-2D ORF numbers (Additional file 1: Table S1), and the inner numbering indicates the loci of the 22 regions of overlapping transcription (ROTs) (for further information, see Additional file 6: Table S3). ROTs with co-occurrence events are shown in bold. ORFs colored in black represent those whose expression profile was not included in the present work (ORFs 64, 83 and 135). **A**. Genomic map relating the physical position of genes to their distribution among the five regulatory modules (communities) of the GRN. **B**. Genomic map showing the gene promoter motifs arrangement in the AgMNPV genome. **C**. Genomic map presenting the genome organization according to gene functions.

Given that some adjacent co-transcribed genes belonging to ROTs grouped in the same community, as expected if these genes shared similar transcription regulation, we investigated if the potential expression of tandem RNA transcripts could influence the regulatory association of nodes in the GRN. We observed that, at a coarse-grain, the bulk of physical distances among AgMNPV-2D genes, expressed in map units, did not show extensive positive correlation with Euclidean distances among expression profiles of each individual ORF (Additional file 7: Figure S4), indicating that the association of gene expression profile is not explained by the tandem organization of ROTs alone. Nevertheless, fifteen of the 37 shared genes among all baculoviruses [12] are within ROTs, shown as asterisks over each physical map in Additional file 6: Figure S3. Moreover, among 56 nodes from community I (Figure 3), 20 belonged to ROTs number 1, 13, 14, 15, 17 and 19 (Additional file 6: Figure S3 and Figure 5A). Thus, one third of genes potentially expressed in tandem were neighbors in the GRN. Furthermore, the two-fold co-occurrence of five community I members in tandem, as observed in ROTs 17, 19 to 21 (in bold in Figure 5) was estimated to be low probability events (*p* = 0.001 each) during Monte Carlo simulations (Additional file 8: Table S4). Among all 45 nodes in the community II, 14 represent genes arranged in pairs throughout the genome (Figure 5A). Nonetheless, just one pair of genes from ROT 2 is probably transcribed in tandem, while ORFs were scattered all over the genome in the communities III, IV and V (Figure 5A), as would be expected by chance (Additional file 8: Table S4). We also found that early, late and unknown promoter motifs (Figure 5B) appear to be interleaved with some degree of heterogeneous distribution in the genome. For example a cluster of 6 ORFs with late motifs in ROTs 10 to 12 (in bold in Figure 5) had a low chance probability (*p* = 0.000735) (Additional file 9: Table S5), while the chance of observing a triple tandem of early and late motifs was higher, (*p* = 0.0211). Moreover, small ORF sets containing similar promoter motifs were arranged contiguously, and most of them coincide with the 22 ROTs we found, which have mostly ORFs with late or early and late promoter motifs. We also investigated how genes involved in the same biological process or with similar functions did cluster in the genome. As shown in Figure 5C, the majority of genes from the same functional category were not adjacent but rather interspersed sense-wise. Nonetheless, the occurrence of tree genes coding for virion proteins in the positive strand in ROTs 19 to 21 had a low chance probability (*p* = 0.00216), while the co-occurrence of 3 genes encoding transcription associated products in the positive strand in ROT 5 (in bold in Figure 5) had a even lower chance probability (*p* = 0.00087) (Additional file 10: Table S6).

## Discussion

### Expression of structural and regulatory genes vary more on less permissive cells

The use of two different infection kinetics allowed us to observe contrasting aspects of the transcriptional program of AgMNPV-2D *in vitro*, providing comprehensive, time-related information on the transcription dynamic and quantitative information on the expression of 149 of its 152 predicted ORFs. The ‘sooner and higher’ expression pattern in UFL-AG-286 compared to IPLB-SF-9 could be explained by the different states of adaptation of the virus to distinct cellular environments, since the infection in IPLB-SF-9 (a less permissive cell line) could be understood as a ‘perturbation’ of the AgMNPV-2D GRN. Nevertheless, we have not noticed significant difference in most genes related with DNA replication, such as *dnapol* (ORF 65), *lef-1* (ORF 19) and *lef-2* (ORF 3) that were expressed at the same time and at similar levels in both cell lines. While replication-associated genes had similar expression, most structural genes had a significant delay in IPLB-SF-9 and notable differences in the magnitude of expression (Additional file 1: Table S1 and Additional file 6: Table S3). Hence, the expression profiles of the structural genes found in IPLB-SF-9 could help explain the delay in viral morphogenesis routinely observed in our laboratory that was also reported for IPLB-SF-21 cells [38].

We consistently observed earlier expression for most of the genes than expected based on the observed for other baculoviruses. For example, the *p6.9* gene (ORF 96) was reported as late, expressed at 12 h p.i. in the AcMNPV [44]. P6.9 is a small protein (6.9 kDa), basic, arginine-rich and it is involved in the condensation of viral DNA. It is not surprising to find this gene expressed earlier in the AgMNPV-2D viral cycle, since DNA replication begins at 2 h p.i. [38] and P6.9 is known to be necessary to package and stabilize the newly synthesized viral genome [45]. In addition, Iwanaga and colleagues [46] also found *p6.9* mRNA and other transcripts earlier in infection, which corroborates our results and earlier observations on other DNA viruses, such as FV-3 and CIV [47-49]. Crucially, the difference we observed could be due to the use of real-time PCR, which is much more efficient and requires fewer targets than hybridization techniques (such as Northern blot), detecting low amounts of RNA initially present in each sampling time. This precocity could simply reflect differences between baculoviruses, given the earlier onset of DNA replication in AgMNPV-2D (2 h p.i.) compared to others, such as AcMNPV (5 h p.i.) [50], SfMNPV (8 h p.i.) [51] and LdMNPV (18 h p.i.) [52].

Furthermore, we also noticed that genes with more than one copy in the genome (such as *iaps*, *pifs* and *ptps*) had distinct expression profiles. For example: (*i*) *iap-3* (ORF 34) was expressed earlier in UFL-AG-286 cells; (*ii*) *iap-2* (ORF 70) simultaneously, whereas; (*iii*) *iap-1* (ORF 40) showed expression only later, in both cell lines, being more expressed than *iap-2* and *iap-3*. Gene duplication and redundancy are important mechanisms that allow adaptive evolution of genomes, and represent 8-20% of the gene content in eukaryotes [53]. We would argue that this allows one of the copies of a gene to evolve with lesser functional constraint [54], which eventually could help it acquiring a new functionality (such as, *iap* and *bro* genes).

### Conserved genes have higher centrality in a modular AgMNPV-2D GRN

By comparing with our randomized models the inferred GRN in UFL-AG-286 cells, where five communities of nodes were identified, it is notable that it had a significantly different modular structure. This was made clear by the trend in both the average connectivity and the average coefficient distribution values for nodes as function of the number of neighbors they had (Additional file 3: Figure S1). By looking at the AgMNPV-2D GRN as a single component we observed that nodes with highest importance, both in terms of BC and SC, included relevant host-interaction functions such as *gp64* (ORF 124) and *egt* (ORF 18). The *gp64* codes for an envelope glycoprotein paramount for effective membrane fusion during the secondary infection that is highly conserved in the group I nucleopolyhedroviruses (NPVs) [55]. Interestingly, the expression of the *egt* gene in *Lymantria dispar* multiple nucleopolyhedrovirus (LdMNPV) was shown to be the genetic basis of climbing behavior and tree top disease in silkworm infected with baculovirus, which facilitates preferential predation of sick larvae while maximizing viral spread in the environment [56].

A relevant property of our inferential procedure was that there was a positive correlation of reduced genetic diversity (θ) and importance of the gene in the GRN, as expected for key components of metabolic networks [7,8]. Core conserved functions also had more temporal consistency of expression and distinct topologic features in the GRN. These coherent results support our modeling effort, since they constitute *independent evidence* for a relationship among phylogenetic data of baculovirus genes and complex GRN attributes inferred from expression data alone.

The observed modular heterogeneity that separates key genes that are functionally related on different communities could help accommodate redundancy and shield important functions, therefore maximizing GRN robustness by compartmentalization [57]. For example, genes possibly associated with anti-apoptotic function (*iap* genes) [58] were well distributed among three communities (I, II and IV), while *dnapol* and *helicase* nested in different communities (III and V). Likewise, assigning several different functions to a given module would also provide robustness by increasing functional redundancy [59]. Nevertheless, some functional associations were evident. Community I was highly populated by structural proteins associated with capsid and virion formation and promoters with early & late motifs (Figure 4). Interestingly, in the AgMNPV-2D 48 of 56 nodes in community I (13 of which are core genes) are conserved in almost all group I alphabaculoviruses sequenced so far. This feature was also observed in community II, in which one third of the nodes consisted of highly conserved genes, many of which were genes associated with DNA replication. The clustering of genes in community II with unidentified regulatory motifs could entail promoter motifs not yet described [60], which is made more compelling giving the high connectivity among its members. Spatially or chemically isolated modules composed of several cellular components and carrying discrete functions are considered fundamental building blocks of cellular organization, but their presence in highly integrated biochemical networks lacks quantitative support. In a key comparative study, the metabolic networks of 43 distinct organisms were shown to have modular organization and in *E. coli*, the hierarchical modularity had a good correlation with metabolic functionality [61]. Spatially or chemically isolated modules composed of several cellular components and carrying discrete functions are considered fundamental building blocks of cellular organization, but their incidence and generality needs to be better investigated across a wider spectrum of organismic complexity, such as in viruses.

Unfortunately, at this time we are not able to wire cell-coded nodes in the AgMNPV-2D GRN. Nevertheless, we would argue that they should have not only a crucial role in the viral cycle, but also should be connected to the real viral GRN [9]. However, viral GRNs encompass both the cellular and viral encoded functions necessary to complete the viral multiplication cycle [9]. Accordingly, preliminary data on RNA suppression subtractive hybridization (SSH) experiments (Oliveira *et al.*, in preparation) indicated that at least 50 cellular distinct genes were down regulated during infection at 24 h.p.i. (Additional file 11: Table S7). By using both Gene Ontology (http://www.geneontology.org/) and EGene (http://www.coccidia.icb.usp.br/egene/) programs while using the eggNOG2.0 database (http://eggnog.embl.de/), we found sequences coding mainly for proteins related to metabolism or protein modification (33.9%), ion transport and energy (12.0%), nucleic acids metabolism (11.3%) and several other functions at a lower frequency. But importantly, we found cellular genes at lower frequency, which are of direct relevance to viral host interaction such as: (*i*) the significantly hypo-expressed anterior fat body protein (AFP), (*ii*) defensin and, (*iii*) heat shock 90 (*hsp90*) (Additional file 11: Table S7). At this juncture, it is hard to assess the extent at which the hypo-expression of cellular genes does not simply reflect cellular degradation due to viral disruption of cellular homeostasis, or is due to interactions with viral gene products. Notwithstanding, these preliminary results could imply that at least 50 additional cellular genes should be wired to the viral GRN for a proper description of the viral life-cycle.

### The conserved genome architecture of Group I baculovirus has implications for gene expression

The presence of highly conserved ROTs in closely related baculoviruses, observed in our sinteny analyses, suggests a complex pattern of gene expression regulation, possibly also present in other baculoviruses [28,31]. The intergenic spacers among ORFs in ROTs are either very short or absent, and overlapping genes are overwhelmingly located in these genomic regions (χ^2^ = 65.041, *p <* 0.00001; d.f. = 1). This would provide a competitive advantage, given that keeping genomes short would speed up genome replication at the cost of maximizing interdependence among superimposed genes [62]. Interestingly, we also found evidence that the virus tries to keep similar attributes dispersed around the genome, mainly avoiding their tandem organization (Additional file 9: Table S5, Additional file 10: Table S6 and Additional file 11: Table S7) with a few notable exceptions. A simple explanation for this observation would be that by avoiding clusters of similar attributes, the genome maximizes robustness of the viral GRN. Moreover, whilst ROTs are somehow conserved among group I NPV, our findings suggest that physical proximity is not a reliable predictor of temporal patterns of gene expression, since in the AgMNPV-2D there was no correlation between the position of genes in the genome and their temporal expression (Additional file 7: Figure S4). These results are in line with the possibility that tandem transcripts from ROTs in baculovirus are not necessarily translated at once [24-26]. Moreover, long mRNAs of several genes have been observed late in infection [27,28,31,63].

The region comprising from ORF 129 (*p24*) to 133 (*alk-exo*) in AcMNPV encodes for diverse tandem transcripts [27], which share the same T-rich 3’ end termination site [64]. These ORFs are transcribed as monocistrons at the onset of infection (up until around 2 h p.i.) and later are transcribed in tandem [27]. The synthesis of long transcripts has been related to regulatory functions, such as promoter occlusion [65] and RNA interference (RNAi) [66,67]. Promoter occlusion is caused by RNA polymerase transcriptional complex activity during the transcription of upstream genes, blocking the monocistronic expression of downstream genes [65]. The expression of antisense sequences in tandem RNAs has been suggested as a mechanism of down-regulation [68,69]. Moreover, it has been assumed that overlapping transcripts should have a functional relevance for regulation of gene expression [27-31]. Possibly, monocistronic expression is sufficiently high as to hamper mRNA detection from tandem transcripts, which would help explaining the lack of correlation shown in Additional file 7: Figure S4. Fittingly, it has been suggested that regulatory elements functioning as ‘internal ribosome entry sites’ (IRES) are functional in the IPLB-SF-9 cell line [70]. Furthermore, the capsid protein VP1054 is probably synthesized by IRES-mediated translation [71], since its monocistronic transcript was not found in AcMNPV and BmNPV gene expression programs [31].

## Conclusions

The transcriptome of the AgMNPV-2D indicated that conserved viral functions showed expression synchronicity, higher betweeness centrality and reduced genetic diversity, consistent with evolutionary constraints present in complex biological systems [7,8]. Most ORFs were expressed at ‘sooner and higher’ levels in a more permissive cell line. In 13 group I alphabaculovirus genomes, we found 22 highly conserved regions of overlapping transcription, whose extended 3’ UTR could play a role in gene expression regulation [66]. The inferred GRN had a modular topology, comprising five communities of highly interconnected nodes, consisting of genes with different functions, promoter motifs, and physical location in the genome. This modular heterogeneity was suggestive of architectural redundancy that could promote robustness [57,59]. The fact that a similar architecture was observed on a simpler GRN, such the one we report for the AgMNPV-2D, tends to support the idea that hierarchical modularity may be an extensive and generic self-similarity feature of system-level biological organization [61].

## Methods

### Primer design and validation

Specific primer pairs for each ORF annotated in the AgMNPV-2D genome [22] were designed using Oligo Analysis Software v. 6.8 (Molecular Biology Insights) (Additional file 12: Table S8). Each pair was tested by PCR amplification and sequencing. Consensus sequences were verified by BLAST searching to check cross-hybridization against each other and the AgMNPV-2D genome. After this initial validation step, each pair of primers was further tested in real-time PCR reactions to ensure its specificity as determined by a unimodal-melting curve. After the specific amplification was confirmed, primer pairs were also validated by five points dilution (10^-2^, 10^-4^, 10^-6^, 10^-8^ and 10^-10^ or 10^-3^, 10^-4^, 10^-5^, 10^-6^ and 10^-7^) amplification by real-time PCR. Reaction efficiency of 0.9 and the correlation coefficient (*r*^2^) values of 0.99 were assumed as minimal quality standards for data acquisition and melting curves were also analyzed to confirm specificity. ORF 135 (*bro-h*) was not included in the study because there were unspecific products for three alternative primer pairs designed inside and upstream of its coding region.

### Cell lines and infection kinetic: virus infection, RNA extraction and reverse transcription

The UFL-AG-286 [39] and IPLB-SF-9 [72] cell lines were maintained in Grace medium (Gibco) supplemented with 10% fetal bovine serum (Gibco). These permissible cell lines were used since AgMNPV has different infection kinetics in them [38]. UFL-AG-286 was derived from embryonic tissue of the lepidopteron *Anticarsia gemmatalis*[39], which is natural host of AgMNPV [19,21,73-76], while IPLB-SF-9 is a clone of IPLB-SF-21 cell line, established from pupal ovaries of *Spodoptera frugiperda,* another Noctuidae species from a different genus [40]. The virus strain AgMNPV-2D [77] was multiplied only in UFL-AG-286 cells, and both cell lines were used in the kinetic infections. Cultures of 10^5^ UFL-AG-286 e IPLB-SF-9 cells were infected at a multiplicity of infection (MOI) of 10 and incubated at 28°C for 1h to allow infection synchronization. Unattached virions were then removed and fresh culture medium was added to the infected cells. Mock-infected samples were treated in the same manner as virus-infected cells, but with fresh culture medium instead of viral suspension. All experiments were done in triplicate. Total RNA was extracted from infected and mock cells at 0, 1, 3, 5, 7, 9, 11, 13, 24 and 48 hp.i. using RNeasy Plus Kit (Qiagen) according to the manufacturer’s recommendations. RNA samples were subjected to DNase I treatments (DNA-free, Ambion) according to the manufacturer’s protocols. 100 ng of each RNA sample was reverse transcribed using both SuperScript III Reverse (Invitrogen) and High-Capacity cDNA Archive kit (Applied Biosystems) using oligo (dT) and random primers (respectively) to allow maximum conversion of mRNA into cDNA. The cDNAs were mixed and used in the real-time PCR reactions.

### Real-time PCR

We used real-time PCR to quantify mRNAs concentration during infection in cell cultures. This was done because of its accurate qualitative and quantitative measurements of the amount of transcripts with high sensibility and reproducibility [78,79], outperforming microarray in these regards [80]. Reactions were performed along with the points of the standard curve and the cDNA samples from kinetic infections, to keep the same conditions for standards and experimental samples. Reactions mixes contained 1.0 μl of cDNA from each time point (or point dilution), 7.5 μl of iQ SYBR Green Supermix kit (Bio-Rad) and 0.5 μM of each primer, in a final volume of 15 μl. The cycling conditions were: 2 min at 96°C, followed by 40 cycles of 30 s at 96°C, 30 s at 50°C /52°C /54°C /57°C (depending on the Tm of primer pair), and 40 s at 72°C. Melting curve analysis was performed increasing the temperature of the last cycle (72°C) until reaching 96°C, 1°C per cycle, 5 s at each cycle. Amplification, detection, and data analysis were performed using the Rotor-Gene 3000 systems (Corbett Life Science).

### Promoter analysis and synteny in regions of overlapping transcription (ROTs)

Promoters were searched in regions comprising 200 bp upstream of the putative start codon of each ORF by screening for known promoter elements. We chose 200 bp since this was determined by several independent studies to harbor all known late promoters (within the first 80 bp upstream from the first ATG) and most known functional early promoters of baculovirus genes. Early promoter (E) indicates a TATA box sequence (TATA or TATAWWW, W= A or T) followed by a CANT motif downstream, transcribed by the host RNA polymerase II [81]. To screen the late promoter motifs transcribed by the viral encoded RNA polymerase, the conserved pattern used was DTAAG [23,82]. Moreover, to investigate the genomic organization of clusters of genes with similar transcriptional profiles that we found in the AgMNPV, we did complete genome overlays including 13 group I alphabaculovirus with the Artemis Comparative Tool [83], while focusing on the conservation of synteny of 22 known regions of overlapping transcription (ROTs) that where first described in AcMNPV [28] and BmNPV [31].

### GRN inference

A conceptual problem inherent to transcriptional data analyses is the assumption that gene expression dynamics is linear, deterministic and constant, while in fact it is mostly non-linear, infrequent and random [84,85]. Expression of genes most often happens as pulses of indeterminate duration [86], and depends on the interplay of different biological components (transcription factors, promoters motifs, RNA polymerases, splicing factors, etc.) at a given temporal (*i.e.*, developmental phase) and spatial (*i.e.*, tissular) context [85]. Moreover, levels of mRNA expression and its encoded protein translation are not necessarily correlated [87], while remarkable differences are observed in mRNA half-life [88]. Notwithstanding, clustering methods were used to analyze baculovirus gene expression transcriptional data [46,60,89]; but because transcript numbers experience iterative exponential fluctuations in time during successive infection cycles, gene expression is better understood as a non-linear system [84,85], taking place on a log-scaled attractor [90-92]. We argue that these properties make simple least-square distance estimates among individual gene expression time-series uninformative and biased, being inadequate for process description [26,93]. Therefore, we used a prediction algorithm for the AgMNPV-2D gene regulatory network that is based on multiple regression and tree-based ensemble methods using our gene expression data (Additional files 1 and 2: Tables S1 and S2) [94]. We chose the method implemented in GENIE3 instead of clustering because it: (*i*) was shown to perform well on reconstructing complex GRNs; (*ii*) does not make any assumption about the nature of gene regulation; (*iii*) infers directed GRNs; (*iv*) can deal with combinatorial and non-linear interactions among genes and; (*v*) provides a confidence ranking of regulatory links. Ranked lists of regulatory links were calculated with GENIE3 loaded in the free software environment for statistical computing and graphics R v.2.10.0 [95], then uploaded the ranked list output in Cytoscape 2.8.0 for network visualization and analysis [96]. To evaluate the importance of node connection in the modeled GRN, we used graph-theoretic methods implemented in the Hub Objects Analyzer (*Hubba*) plug-in for Cytoscape, which were shown for example to recover known yeast interactome data with greater than 70% accuracy [97]. With *Hubba*, we used Maximum Neighborhood Component (MNC) and Density of Maximum Neighborhood Component (DMNC) algorithms. This approach was helpful to obtain a minimal set of most important edges necessary to fully connect all viral nodes in the GRN.

### Measuring complex network attributes

Modular networks have subsets of nodes that are densely connected within and weekly connected between subsets [61,98]. Therefore, betweenness centrality (BC), which estimates the number of shortest paths from all vertices to all others that pass through a node, is a more informative measure of topologic importance of a node than connectivity (*k*), which indicates the number of connections of a node irrespective of its neighbor’s connectivity properties. Likewise, we also estimated other relevant topologic attributes that inform on network architecture such as: (*i*) the average clustering coefficient distribution (ACC) that gives the average of the clustering coefficients for all nodes with all quantities of neighbors, helping to find modularity in networks; (*ii*) the average neighborhood connectivity (ANC) that is the average connectivity of all neighbors of a node, which also informs on the sub-structuring of a network; (*iii*) stress centrality (SC) [99,100] that is the number of shortest paths passing through a node and; (*iv*) modularity (*Q*), which measures the number of edges falling within groups minus the expected number in an equivalent network with edges placed at random, quantifying the level of network compartmentalization in communities of nodes. While comparing genomic and GRN organizations we also investigated if three attributes (GRN communities, promoter motifs and functional categories) were spatially organized in the AgMNPV-2D. Hence, we estimated their probability of arbitrary co-occurrence by a Monte Carlo (MC) procedure, in which the attributes were randomized 100 million times without replacement, using a PERL script available from the authors upon request. During our randomization exercises, the co-occurrences were incremented when an attribute randomly sampled from the list was identical to the previously sampled. Thus, the chance to find co-occurrence events of an attribute depends on its frequency in the list. During MC simulations this approach should introduce compositional biases, since it does not reflect the real gene accretion process during baculovirus genome evolution [101] but critically, it does generate virtual attribute arrangements with the same total number of components as the real genome.

### Estimating the evolutionary change on each AgMNPV-2D ORF

We also sought to generate node attributes such as molecular evolution (*i.e.*, genetic diversity), to correlate with the estimated network parameters. To verify this property, we inferred the genetic diversity (θ) in a comparative set of 121 coding sequences shared among 4 group I alphabaculoviruses: AgMNPV (*Anticarsia gemmatalis* MNPV, DQ813662), CfDEFNPV (*Choristoneura fumiferana* defective NPV, AY327402), EppoMNPV (*Epiphyas postvittana* MNPV, AY043265) and CfMNPV (*Choristoneura fumiferana* MNPV, AF512031). These baculoviruses were chosen due to their close phylogenetic relationship, and the homologues ORFs were selected according to Oliveira *et al.*[22]. Nucleotide sequences were extracted from GenBank database using the annotation software Artemis [102]. Multiple sequence alignments for each gene were generated using ClustalW [103] and manually adjusted using Bioedit [104]. Finally, we used a phylogeny-based Markov Chain Monte Carlo (MCMC) Bayesian method implemented in the Coalesce 1.5 beta program [105] to estimate the genetic diversity (*θ* = *Ne*.*μ*) for each viral gene alignment and compared *θ* to betweenness centrality (BC) estimates obtained for ‘core’ (replication and capsid) and ‘satellite’ (all the others) functions [101]. With Coalesce, an initial Watterson estimate of θ was used and the MCMC consisted of 10 short chains and 2 long chains of 4000 steps each, during which the viral trees for each gene were optimized and θ estimated.

## Competing interests

The authors declare that they have no competing interests. The funders had no role in study design, data collection and analysis, decision to publish, or preparation of the manuscript.

## Authors’ contributions

JVCO and PMAZ designed the experiments, analyzed the data and wrote the manuscript. AFB, CTB and CCMF analyzed the data and wrote the manuscript. AI edited the manuscript. All authors read and approved the final manuscript.

## Supplementary Material

Additional file 1: Table S1 Showing the temporal expression of AgMNPV-2D genes in UFL-AG-286 cell line. Click here for file

Additional file 2: Table S2 Showing the temporal expression of AgMNPV-2D genes in IPLB-SF-9 cell line. Click here for file

Additional file 3: Figure S1 Plotting the average clustering coefficient distribution and average neighborhood connectivity of the AgMNPV-2D GRN in both cell lines compared to random data. Click here for file

Additional file 10: Table S6 Showing chance probabilities of Monte Carlo simulation to sample the co-occurrences of functional categories. Click here for file

Additional file 4: Figure S2 Depicting the comparison of betweeness centrality (BC) and genetic diversity (θ) values among core and satellite genes. Click here for file

Additional file 5: Figure S3 Depicting a syntenic map of 13 sequenced group I alphabaculovirus genomes showing the regions of overlapping transcription (ROTs) and their relative position. Click here for file

Additional file 6: Table S3 Presenting the conserved open reading frames located in putative regions of overlapping transcription of 13 group I alphabaculoviruses genomes. Click here for file

Additional file 7: Figure S4 A chart that shows a plot of the Euclidean distances in expression profiles and the physical distance among genes in the viral genome. Click here for file

Additional file 8: Table S4 Showing chance probabilities of Monte Carlo simulation to sample the co-occurrences of GRN communities. Click here for file

Additional file 9: Table S5 Showing chance probabilities of Monte Carlo simulation to sample the co-occurrences of promoter motifs. Click here for file

Additional file 11: Table S7 Listing the set of cellular genes from UFL-AG-286 cell line that were detected by RNA suppression subtractive hybridization (SSH) during a 24 h.p.i. AgMNPV-2D infection. Click here for file

Additional file 12: Table S8 Which lists the primer pairs designed to amplify and quantify the expression of AgMNPV-2D ORFs. Click here for file
