# Supplementary material for: Modularity and evolutionary constraints in a baculovirus gene regulatory network
Source: BMC Syst Biol. 2013 Sep 4;7:87. doi: 10.1186/1752-0509-7-87 (PMC3879405; doi:10.1186/1752-0509-7-87)
Supplement: Additional file 3: Figure S1 — Plotting the average clustering coefficient distribution and average neighborhood connectivity of the AgMNPV-2D GRN in both cell lines compared to random data. [file 1752-0509-7-87-S3.xls]

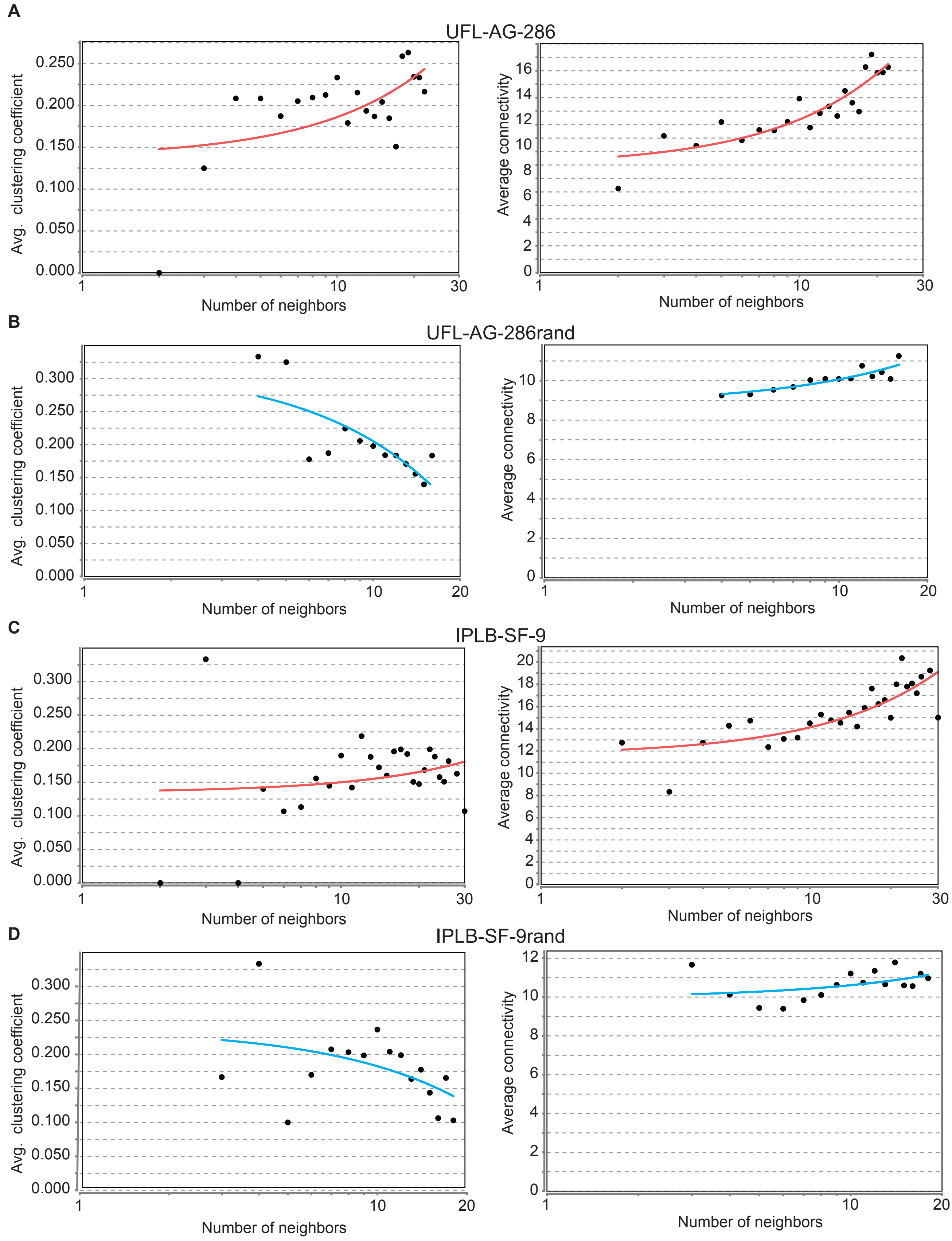

Figure S1. Topological features of the AgMNPV-2D GRN in both cell lines (in red) compared to random data (in blue). **A**. Average clustering coefficient distribution (ACC) and Average neighborhood connectivity (ANC) versus the number of neighbors for each and every node of networks generated in UFL-AG-286 cells. **B**. ACC and ANC for the randomized UFL-AG-286 data. **C**. ACC and ANC for the IPLB-SF-9 infection. **D**. ACC and ANC for the randomized IPLB-SF-9 data. Since the average clustering coefficient distribution informed on the sub-structuring of a network, the data indicated that real data, with increasing ACC as the number of neighbors increase compared with random networks, have more modularity and hierarchical structure than that expected by chance. This effect was more pronounced in the UFL-AG-286 infection than in IPLB-SF-9 infection, which may reflect adaptive features of the virus to its original host. ANC distributions were significantly different in real and scrambled data. Real data had higher number of neighbors with higher ANC with positive increase of ANC as the number of neighbors increased. Like for ACC, ANC also indicates a noticeable difference among real and random data. Real data have larger number of groups (indicated by the higher number of neighbors per node) with higher connectivity, which indicates that real GRN have more structured hierarchical structures.
