# Supplementary material for: Modularity and evolutionary constraints in a baculovirus gene regulatory network
Source: BMC Syst Biol. 2013 Sep 4;7:87. doi: 10.1186/1752-0509-7-87 (PMC3879405; doi:10.1186/1752-0509-7-87)
Supplement: Additional file 10: Table S6 — Showing chance probabilities of Monte Carlo simulation to sample the co-occurrences of functional categories. [file 1752-0509-7-87-S10.docx]

**Table S6.** Chance probabilities of Monte Carlo simulation to sample the co-occurrences of functional categories.

| **Functions**  **Co-occurrences** | **Auxiliary** | | **Capsid** | | **Modulation** | | **Replicon** | | **Transcription** | | **Unknown** | | **Virion** | | **NA*** | |
| --- | --- | --- | --- | --- | --- | --- | --- | --- | --- | --- | --- | --- | --- | --- | --- | --- |
|  | -^1^ | +^2^ | - | + | - | + | - | + | - | + | - | + | - | + | - | + |
| 2 | 2.34E-2 | 3.89E-2 | 3.93E-3 | 6.19E-3 | 8.01E-3 | 3.17E-4 | 5.80E-3 | 4.48E-3 | 3.92E-3 | 1.03E-2 | 7.49E-2 | 6.41E-2 | 1.98E-2 | 1.82E-2 | 0 | 3.13E-4 |
| 3 | 3.17E-3 | 7.89E-3 | 1.54E-4 | 3.61E-4 | 5.38E-4 | 0 | 3.09E-4 | 2.07E-4 | 1.55E-4 | 8.70E-4 | 2.12E-2 | 1.66E-2 | 2.39E-3 | 2.16E-3 | 0 | 0 |
| 4 | 3.25E-4 | 8.35E-4 | 4.59E-6 | 1.67E-5 | 1.81E-5 | 0 | 1.14E-5 | 8.16E-6 | 2.00E-6 | 4.56E-5 | 5.47E-3 | 4.37E-3 | 2.34E-4 | 2.31E-4 | 0 | 0 |
| 5 | 3.14E-5 | 1.39E-4 | 1.00E-7 | 6.80E-7 | 1.14E-6 | 0 | 2.40E-7 | 1.70E-7 | 5.00E-8 | 2.81E-6 | 1.36E-3 | 8.92E-4 | 2.34E-5 | 2.32E-5 | 0 | 0 |
| 6 | 3.59E-6 | 2.10E-5 | 1.00E-8 | 1.00E-8 | 5.00E-8 | 0 | 1.00E-8 | 0 | 0 | 2.30E-7 | 3.08E-4 | 2.04E-4 | 2.10E-6 | 2.03E-6 | 0 | 0 |
| 7 | 3.60E-7 | 3.59E-6 | 0 | 0 | 0 | 0 | 0 | 0 | 0 | 0 | 7.79E-5 | 5.00E-5 | 1.70E-7 | 1.70E-7 | 0 | 0 |
| 8 | 4.00E-8 | 5.80E-7 | 0 | 0 | 0 | 0 | 0 | 0 | 0 | 0 | 1.80E-5 | 1.04E-5 | 0 | 2.00E-8 | 0 | 0 |
| 9 | 1.00E-8 | 4.00E-8 | 0 | 0 | 0 | 0 | 0 | 0 | 0 | 0 | 4.51E-6 | 2.41E-6 | 0 | 0 | 0 | 0 |
| 10 | 0 | 2.00E-8 | 0 | 0 | 0 | 0 | 0 | 0 | 0 | 0 | 1.46E-6 | 5.10E-7 | 0 | 0 | 0 | 0 |
| 11 | 0 | 1.00E-8 | 0 | 0 | 0 | 0 | 0 | 0 | 0 | 0 | 3.50E-7 | 1.10E-7 | 0 | 0 | 0 | 0 |
| 12 | 0 | 0 | 0 | 0 | 0 | 0 | 0 | 0 | 0 | 0 | 9.00E-8 | 4.00E-8 | 0 | 0 | 0 | 0 |
| 13 | 0 | 0 | 0 | 0 | 0 | 0 | 0 | 0 | 0 | 0 | 4.00E-8 | 3.00E-8 | 0 | 0 | 0 | 0 |
| 14 | 0 | 0 | 0 | 0 | 0 | 0 | 0 | 0 | 0 | 0 | 1.00E-8 | 0 | 0 | 0 | 0 | 0 |
| 15 | 0 | 0 | 0 | 0 | 0 | 0 | 0 | 0 | 0 | 0 | 1.00E-8 | 0 | 0 | 0 | 0 | 0 |

*These genes were not uncovered by real time PCR reactions.

^1^Negative strand of viral DNA.

^2^Positive strand of viral DNA.
