# Supplementary material for: Modularity and evolutionary constraints in a baculovirus gene regulatory network
Source: BMC Syst Biol. 2013 Sep 4;7:87. doi: 10.1186/1752-0509-7-87 (PMC3879405; doi:10.1186/1752-0509-7-87)
Supplement: Additional file 4: Figure S2 — Depicting the comparison of betweeness centrality (BC) and genetic diversity (θ) values among core and satellite genes. [file 1752-0509-7-87-S4.pdf]

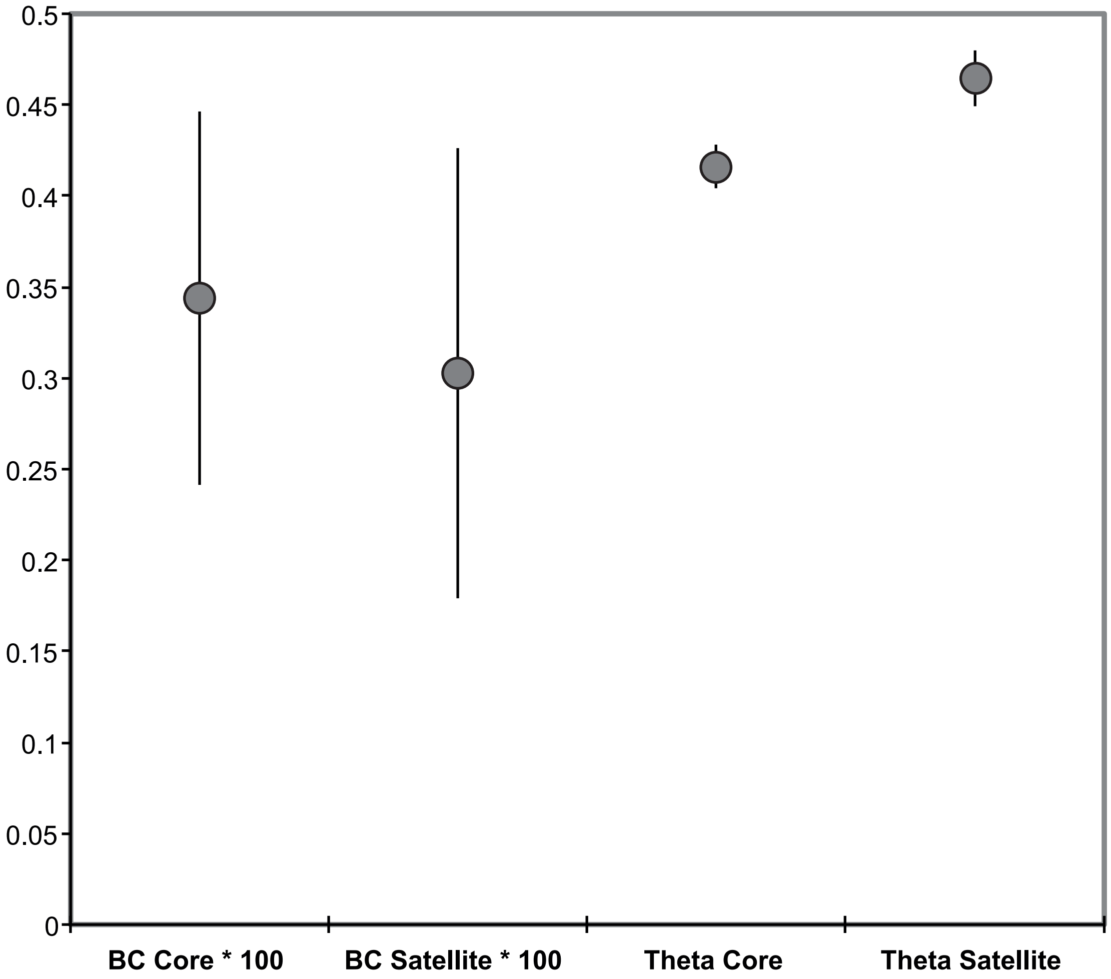

Figure S2. Comparison of 74 genes with capsid and replication-associated functions, compared to the 75 other genes, grouped as ‘core’ or ‘satellite’ respectively (Zanotto & Krakauer, 2008). The median values of betweeness centrality (BC) and genetic diversity (θ) and the associated standard errors are shown. BC values were multiplied by 100 for comparative scaling with θ.
