# Supplementary material for: Modularity and evolutionary constraints in a baculovirus gene regulatory network
Source: BMC Syst Biol. 2013 Sep 4;7:87. doi: 10.1186/1752-0509-7-87 (PMC3879405; doi:10.1186/1752-0509-7-87)
Supplement: Additional file 5: Figure S3 — Depicting a syntenic map of 13 sequenced group I alphabaculovirus genomes showing the regions of overlapping transcription (ROTs) and their relative position. [file 1752-0509-7-87-S5.pdf]

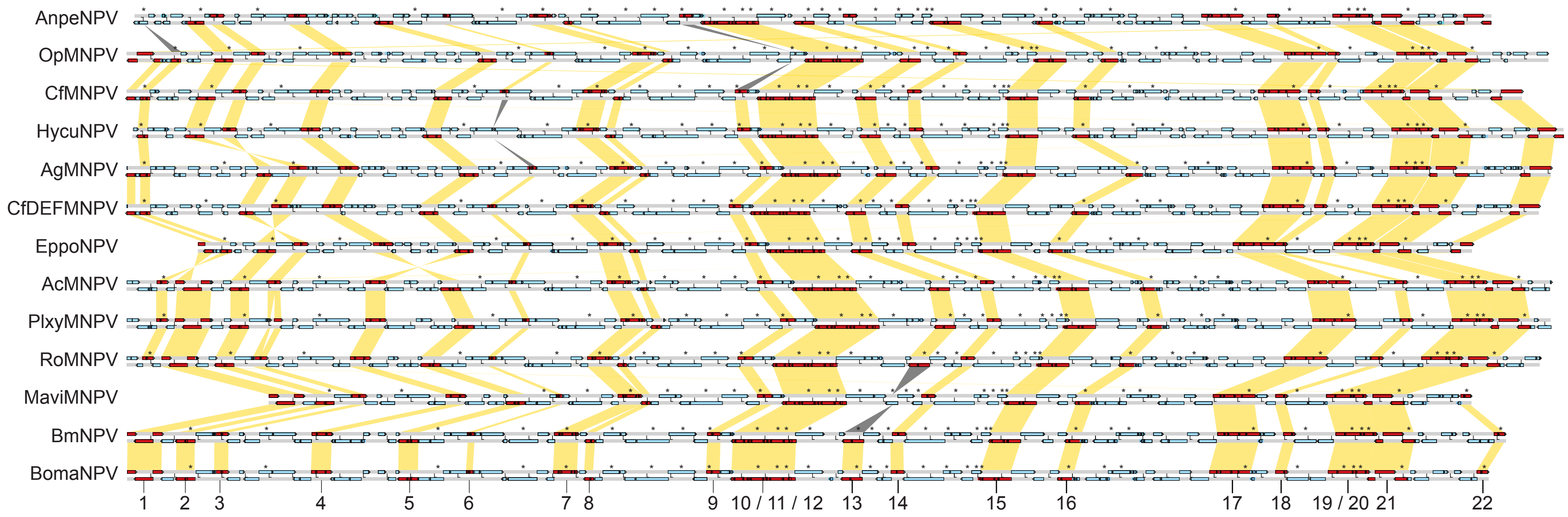


**Figure S3.** Syntenic map of 13 sequenced group I alphabaculovirus genomes. Genomes are shown in a linear fashion, and their gene order is presented according to their GenBank files (except for AnpeNPV and HycuNPV, whose genomes were inverted). The genomic segments colored in red indicate regions of overlapping transcription (ROTs) conserved among 13 group I alphabaculoviruses. The yellow stripes connecting the genomes highlight the relative position of each ROT in different genomes (see ROT numbering in the bottom of the figure). Inversions in genomic segments are indicated by twisted stripes, and black triangular stripes point towards genomic regions in which were detected the absence of a specific ORF, as observed in AnpeNPV, OpMNPV, HycuNPV and MaviMNPV. Asterisks (*) indicate the position of the 31 shared genes in the genomes. For further detailed information, see Table S3.
