# Supplementary material for: Modularity and evolutionary constraints in a baculovirus gene regulatory network
Source: BMC Syst Biol. 2013 Sep 4;7:87. doi: 10.1186/1752-0509-7-87 (PMC3879405; doi:10.1186/1752-0509-7-87)
Supplement: Additional file 7: Figure S4 — A chart that shows a plot of the Euclidean distances in expression profiles and the physical distance among genes in the viral genome. [file 1752-0509-7-87-S7.pdf]

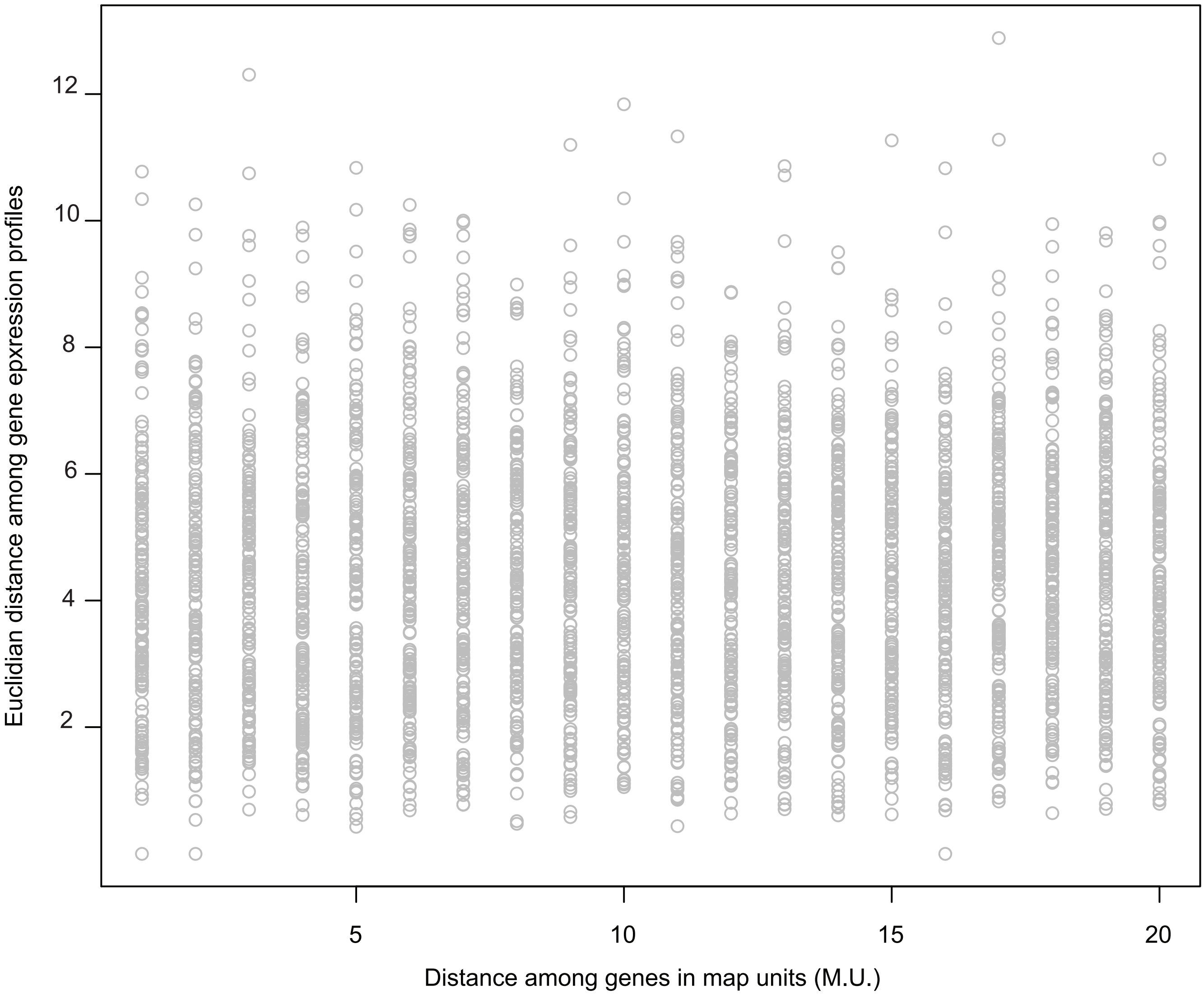

Figure S4. Plot of the Euclidean distances in expression profiles and physical distance among genes in the viral genome in map units. There is no significant correlation between these two variables indicating that the physical proximity of ORFs does not explain their temporal expression pattern.
