# Supplementary material for: Modularity and evolutionary constraints in a baculovirus gene regulatory network
Source: BMC Syst Biol. 2013 Sep 4;7:87. doi: 10.1186/1752-0509-7-87 (PMC3879405; doi:10.1186/1752-0509-7-87)
Supplement: Additional file 8: Table S4 — Showing chance probabilities of Monte Carlo simulation to sample the co-occurrences of GRN communities. [file 1752-0509-7-87-S8.docx]

**Table S4.** Chance probabilities of Monte Carlo simulation to sample the co-occurrences of GRN communities.

| **GRN**  **Communities**  **Co-occurrences** | **I** | | **II** | | **III** | | **IV** | | **V** | | **NA*** | |
| --- | --- | --- | --- | --- | --- | --- | --- | --- | --- | --- | --- | --- |
|  | -^1^ | +^2^ | - | + | - | + | - | + | - | + | - | + |
| 2 | 8.52E-2 | 3.17E-4 | 7.48E-2 | 5.57E-2 | 1.98E-2 | 4.31E-2 | 8.00E-3 | 6.20E-3 | 1.21E-3 | 0 | 0 | 3.17E-4 |
| 3 | 2.67E-2 | 0 | 2.25E-2 | 1.32E-2 | 2.40E-3 | 8.60E-3 | 5.28E-4 | 3.53E-4 | 1.53E-5 | 0 | 0 | 0 |
| 4 | 7.82E-3 | 0 | 4.42E-3 | 2.60E-3 | 2.37E-4 | 1.46E-3 | 2.68E-5 | 1.33E-5 | 0 | 0 | 0 | 0 |
| 5 | 1.99E-3 | 0 | 1.14E-3 | 5.39E-4 | 1.93E-5 | 2.33E-4 | 1.29E-6 | 5.60E-7 | 0 | 0 | 0 | 0 |
| 6 | 5.73E-4 | 0 | 3.16E-4 | 1.15E-4 | 1.47E-6 | 4.06E-5 | 4.00E-8 | 3.00E-8 | 0 | 0 | 0 | 0 |
| 7 | 1.42E-4 | 0 | 7.58E-5 | 2.48E-5 | 1.90E-7 | 5.84E-6 | 0 | 0 | 0 | 0 | 0 | 0 |
| 8 | 3.87E-5 | 0 | 1.82E-5 | 4.67E-6 | 3.00E-8 | 9.40E-7 | 0 | 0 | 0 | 0 | 0 | 0 |
| 9 | 1.08E-5 | 0 | 4.01E-6 | 8.90E-7 | 0 | 2.00E-7 | 0 | 0 | 0 | 0 | 0 | 0 |
| 10 | 3.14E-6 | 0 | 1.20E-6 | 2.00E-7 | 0 | 2.00E-8 | 0 | 0 | 0 | 0 | 0 | 0 |
| 11 | 9.20E-7 | 0 | 3.50E-7 | 5.00E-8 | 0 | 0 | 0 | 0 | 0 | 0 | 0 | 0 |
| 12 | 2.40E-7 | 0 | 1.10E-7 | 0 | 0 | 0 | 0 | 0 | 0 | 0 | 0 | 0 |
| 13 | 4.00E-8 | 0 | 4.00E-8 | 0 | 0 | 0 | 0 | 0 | 0 | 0 | 0 | 0 |
| 14 | 0 | 6.00E-8 | 0 | 0 | 0 | 0 | 0 | 0 | 0 | 0 | 0 | 0 |
| 15 | 1.00E-8 | 0 | 0 | 0 | 0 | 0 | 0 | 0 | 0 | 0 | 0 | 0 |
| 16 | 2.00E-8 | 0 | 0 | 0 | 0 | 0 | 0 | 0 | 0 | 0 | 0 | 0 |

*These genes were not uncovered by real time PCR reactions.

^1^Negative strand of viral DNA.

^2^Positive strand of viral DNA.
