# Supplementary material for: Modularity and evolutionary constraints in a baculovirus gene regulatory network
Source: BMC Syst Biol. 2013 Sep 4;7:87. doi: 10.1186/1752-0509-7-87 (PMC3879405; doi:10.1186/1752-0509-7-87)
Supplement: Additional file 9: Table S5 — Showing chance probabilities of Monte Carlo simulation to sample the co-occurrences of promoter motifs. [file 1752-0509-7-87-S9.docx]

**Table S5.** Chance probabilities of Monte Carlo simulation to sample the co-occurrences of promoter motifs.

| **Promoters**  **Co-occurrences** | **Early** | | **Late** | | **Early and Late** | | **Unknown** | | **NA*** | |
| --- | --- | --- | --- | --- | --- | --- | --- | --- | --- | --- |
|  | -^1^ | +^2^ | - | + | - | + | - | + | - | + |
| 2 | 3.53E-2 | 6.20E-3 | 9.48E-2 | 5.98E-2 | 3.53E-2 | 7.33E-2 | 2.33E-2 | 5.56E-2 | 0 | 3.10E-4 |
| 3 | 6.51E-3 | 3.62E-4 | 3.46E-2 | 1.48E-2 | 6.38E-3 | 2.11E-2 | 3.28E-3 | 1.37E-2 | 0 | 0 |
| 4 | 8.57E-4 | 1.30E-5 | 1.01E-2 | 3.45E-3 | 1.01E-3 | 5.37E-3 | 3.38E-4 | 2.43E-3 | 0 | 0 |
| 5 | 1.54E-4 | 5.40E-7 | 2.68E-3 | 7.72E-4 | 1.46E-4 | 1.30E-3 | 3.13E-5 | 4.62E-4 | 0 | 0 |
| 6 | 2.41E-5 | 4.00E-8 | 7.35E-4 | 1.68E-4 | 2.22E-5 | 3.28E-4 | 3.80E-6 | 9.17E-5 | 0 | 0 |
| 7 | 3.76E-6 | 0 | 2.17E-4 | 3.13E-5 | 3.16E-6 | 8.85E-5 | 4.10E-7 | 1.67E-5 | 0 | 0 |
| 8 | 4.80E-7 | 0 | 7.04E-5 | 7.92E-6 | 4.00E-7 | 2.10E-5 | 5.00E-8 | 3.13E-6 | 0 | 0 |
| 9 | 6.00E-8 | 0 | 2.14E-5 | 1.47E-6 | 3.00E-8 | 5.55E-6 | 0 | 7.10E-7 | 0 | 0 |
| 10 | 1.00E-8 | 0 | 6.41E-6 | 2.60E-7 | 1.00E-8 | 1.09E-6 | 0 | 1.90E-7 | 0 | 0 |
| 11 | 0 | 0 | 2.28E-6 | 1.10E-7 | 0 | 3.50E-7 | 0 | 3.00E-8 | 0 | 0 |
| 12 | 0 | 0 | 7.20E-7 | 3.00E-8 | 0 | 7.00E-8 | 0 | 0 | 0 | 0 |
| 13 | 0 | 0 | 2.20E-7 | 0 | 0 | 2.00E-8 | 0 | 0 | 0 | 0 |
| 14 | 0 | 0 | 7.00E-8 | 0 | 0 | 0 | 0 | 1.00E-8 | 0 | 0 |
| 15 | 0 | 0 | 1.00E-8 | 0 | 0 | 0 | 0 | 0 | 0 | 0 |
| 16 | 0 | 0 | 1.00E-8 | 0 | 0 | 0 | 0 | 0 | 0 | 0 |

*These genes were not uncovered by real time PCR reactions.

^1^Negative strand of viral DNA.

^2^Positive strand of viral DNA.
